# Valency based novel quantitative structure property relationship (QSPR) approach for predicting physical properties of polycyclic chemical compounds

**DOI:** 10.1038/s41598-024-54962-5

**Published:** 2024-03-25

**Authors:** Ali Raza, Mishal Ismaeel, Fikadu Tesgera Tolasa

**Affiliations:** 1 Department of Mathematics, University of Punjab Lahore, Lahore, Pakistan; 2 Department of Mathematics, Nanjing University of Science and Technology, Nanjing, China; 3 Department of Mathematics, Dambidollo University, Dembidollo, Oromia Ethiopia

**Keywords:** Topological descriptor, Regression models, Neighborhood degree, Nanosheets, Drug delivery, Medicinal chemistry, Pharmaceutics, Drug discovery, Materials science, Mathematics and computing

## Abstract

In this study, we introduce a novel valency-based index, the neighborhood face index (NFI), designed to characterize the structural attributes of benzenoid hydrocarbons. To assess the practical applicability of NFI, we conducted a linear regression analysis utilizing numerous physiochemical properties associated with benzenoid hydrocarbons. Remarkably, the results revealed an extraordinary correlation exceeding 0.9991 between NFI and these properties, underscoring the robust predictive capability of the index. The NFI, identified as the best-performing descriptor, is subsequently investigated within certain infinite families of carbon nanotubes. This analysis demonstrates the index’s exceptional predictive accuracy, suggesting its potential as a versatile tool for characterizing and predicting properties across diverse molecular structures, particularly in the context of carbon nanotubes.

## Introduction

Exploring the correlation between the molecular structures and numerical attributes of biological, physical, and chemical properties across diverse compounds stands as a notable application of chemical graph theory. This has led to the introduction of qualitative structure–property relationships (QSPR) and qualitative structure-activity relationships (QSAR). Within QSPR/QSAR studies, topological indices (TIs) emerge as fundamental tools, serving as numeric graph invariants that establish connections between molecular structures and the bio-physical properties of chemical compounds^1–5^. Essentially, TIs function as transformations assigning positive real numbers to graphs. These numerical descriptors play a crucial role in investigating boiling points, melting points, bond energies, and intermolecular forces in existing compounds. Moreover, TIs contribute to predicting the physical properties of different chemical compounds under development, enabling the design of compounds with desired physio-chemical and biochemical characteristics. This streamlined approach reduces additional costs and time, addressing a significant challenge, particularly in developing countries. Extensive research has been conducted on TIs and their applications, as detailed in^6–9^.

Now we define some notations and preliminaries before proceeding further with the study of a specific index. In the literature, notations of vertices, edges, and faces of a planar graph are well defined. For the notions and notations not given here, we refer^10^ to the readers. Degree of a vertex $\nu$ is denoted by $d(\nu )$ while neighborhood degree is denoted by $d_n(\nu )$. Consider a molecular graph *G*(*V*, *E*) of a molecular compound, where *E*, set of edges, represents bond among the atoms and *V* is set of vertices represents the atoms. Face is a region bounded by some vertices *v* and sum of degree of these incident to particular face is known as degree of face i.e. $d(\pounds ) = \sum _{v \sim \pounds }d(v)$. Similarly, neighborhood degree of face is calculated by adding neighborhood degrees of all incident vertex i.e. $d_n(\pounds ) = \sum _{v \sim \pounds }d_n(v)$. Different structure descriptors (TIs) can be evaluated by using vertices, edges or both. The vertex connectivity index *RCI*(*G*) and edge connectivity index *ECI*(*G*) were introduced by Randic^11^ and Estrada^12^ respectively as:

$$\begin{aligned} RCI (G)&=\sum _{u\nu \in G}\left[ d_u \times d_{\nu }\right] ^{-\frac{1}{2}}\\ ECI(G)&=\sum _{e \sim f}\left[ d_e \times d_f\right] ^{-\frac{1}{2}}. \end{aligned}$$

Nikolic and Trinajstic^13^ compared these indices for benzenoid hydrocarbons and model equations to predict bond energies and boiling points of hydro-benzenoid with error range of 0.8–2%. To increase the efficiency of their regression equations, Jamil et al.^14^ introduced a new topological index, known as the Face Index .

**Figure 1:**
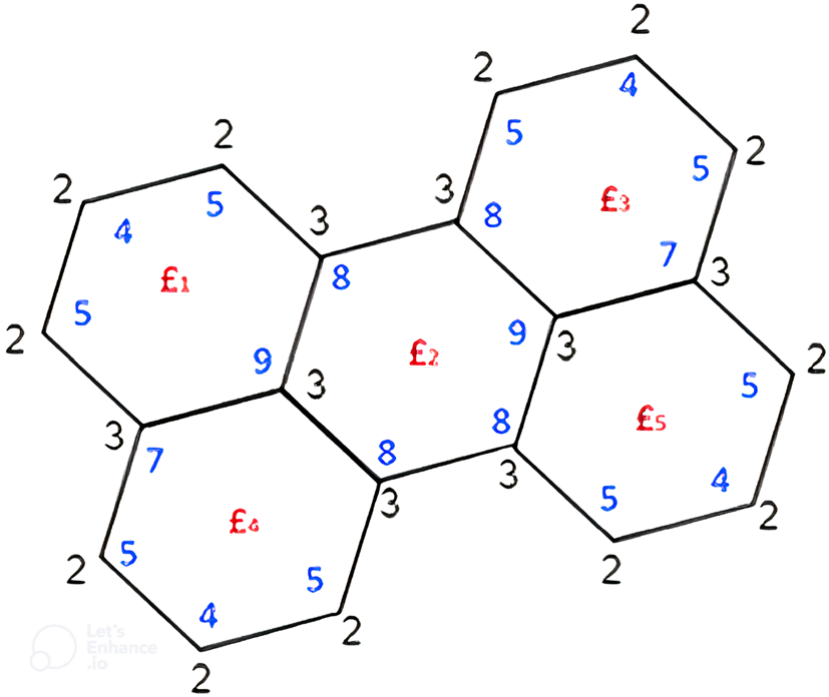
Vertices degree of the perylene benzenoid graph.

Motivated by the above work, we introduced a new topological index namely Neighborhood Face Index , denoted by *NFI*, and defined as:

$$\begin{aligned} NFI(G)=\sum _{\pounds \in F(G)}d_n(\pounds )=\sum _{ \pounds \in F(G)}d_n(\nu ) \ \ \ \hbox { where }\nu \sim \pounds . \end{aligned}$$

which exhibit good correlation with numerous physical properties like bond energies and boiling points along-with stronger prediction ability for benzenoid hydrocarbons. Consider the two dimensional graph of perylene benzenoid graph as shown in Fig. 1 where vertices degree (black), neighbourhood degree (blue) and different internal faces (red) are mentioned. By adding the neighbourhood degree of the vertices which are adjacent to a particular face, we obtain $d_n(\pounds _1)=38, \ d_n(\pounds _2)=50, \ d_n(\pounds _3)=38, \ d_n(\pounds _4)=38, \ d_n(\pounds _5)=38$ and the external face $\pounds _{\infty }$ has face degree 102. Then by definition, $NFI=\sum d_n(\pounds )=304$.

Nanotechnology is catalyzing a revolution in the 21st century, impacting various fields such as space exploration, entertainment, and communication through the creation of innovative materials and devices. Carbon nanotubes, in particular, are on the verge of replacing traditional electronic materials, aiming to construct smaller, faster, and more efficient devices and microchips. The realm of nanotechnology has seen the introduction of diverse nanostructures, including nanocages, magnetic nanochains, nanosheets, nanofibers, and quantum heterostructures. These tubular nanostructures exhibit unique mechanical properties attributed to their stiff and elastic nature^15–17^. Carbon nanotorus structures, known for their multi-layered and caged configurations, find widespread applications in electronics and magnetism^18–20^. Additionally, the extensive use of nanosheets in home appliances and industry is briefly discussed in existing literature^21–25^.

In the present study, we explore regression models involving the neighborhood face index (NFI) in conjunction with various structural parameters such as the randic index, edge connectivity index, $\pi$-electron energy, and boiling points of hydrobenzenoids. Precise formulas for graphene, $C_4 C_8 (S)$, $C_4 C_8 (R)$, and H-Naphthalenic Nanosheets are derived. The computational aspects of our work involve the use of Matlab for mathematical calculations and verifications, Maple for graphical analysis and plotting of results, and ChemSketch for drawing molecular graphs. Physio-chemical properties of benzenoid hydrocarbons are detailed in Table 1, providing exact values for neighborhood face index, randic index, edge connectivity index, $\pi$-electron energy, and boiling points of 21 common benzenoid hydrocarbons. Experimental values for $\pi$-electron energy and boiling points are extracted from previous literature^26,27^. Leveraging the data in Table 1, we establish regression models for the newly introduced neighborhood face index and discuss its chemical applicability.

**Table 1:** Neighborhood face index, vertex connectivity index, edge connectivity index, $\pi -$energy and boiling points of benzenoid hydrocarbons.

Benzenoid hydrocarbons	Neighborhood face index	Randic index	Connectivity edge index	$\pi$ electron energy	Boiling points
Benzene	48	3.00	3.0000	8.0000	80.10
Naphthalene	114	4.97	5.4550	13.6830	218.2
Phenanthrene	182	6.96	7.9260	19.4480	338.1
Anthracene	180	6.93	7.9420	19.3140	340.3
Chrysene	250	8.93	10.2470	25.1920	431.4
Benzanthracene	248	8.92	10.4140	25.1010	424.9
Triphenylene	255	8.96	10.4140	25.2750	428.9
Tetracene	246	8.89	10.4300	25.1880	440.1
Benzo(a)pyrene	302	9.92	11.8970	28.2200	496.2
Benzo(e)pyrene	304	9.93	11.8970	28.3360	493.1
Perylene	304	9.93	11.8970	28.2450	497.4
Anthanthrene	354	10.89	13.3970	31.2530	547.3
Benzoperylene	356	10.92	13.3790	31.4250	542.2
Dibenzo(a,c)anth	318	10.92	12.9020	30.9250	535.1
Dibenzo(a,h)anth	316	10.89	12.8850	30.8810	535.0
Dibenzo(a,i)anth	316	10.89	13.2180	30.8800	531.0
Picene	318	10.92	12.6860	30.9430	519.0
Coronene	408	11.89	14.8630	34.5720	590.1
Dibenzo(a,h)pyr	370	11.57	14.3850	33.9280	596.2
Dibenzo(a,g)pyr	372	11.49	14.3850	33.9540	594.3
Pyrene	234	7.93	9.4080	22.5060	393.1

### Linear regression model between NFI, RI and ECI

**Figure 2:**
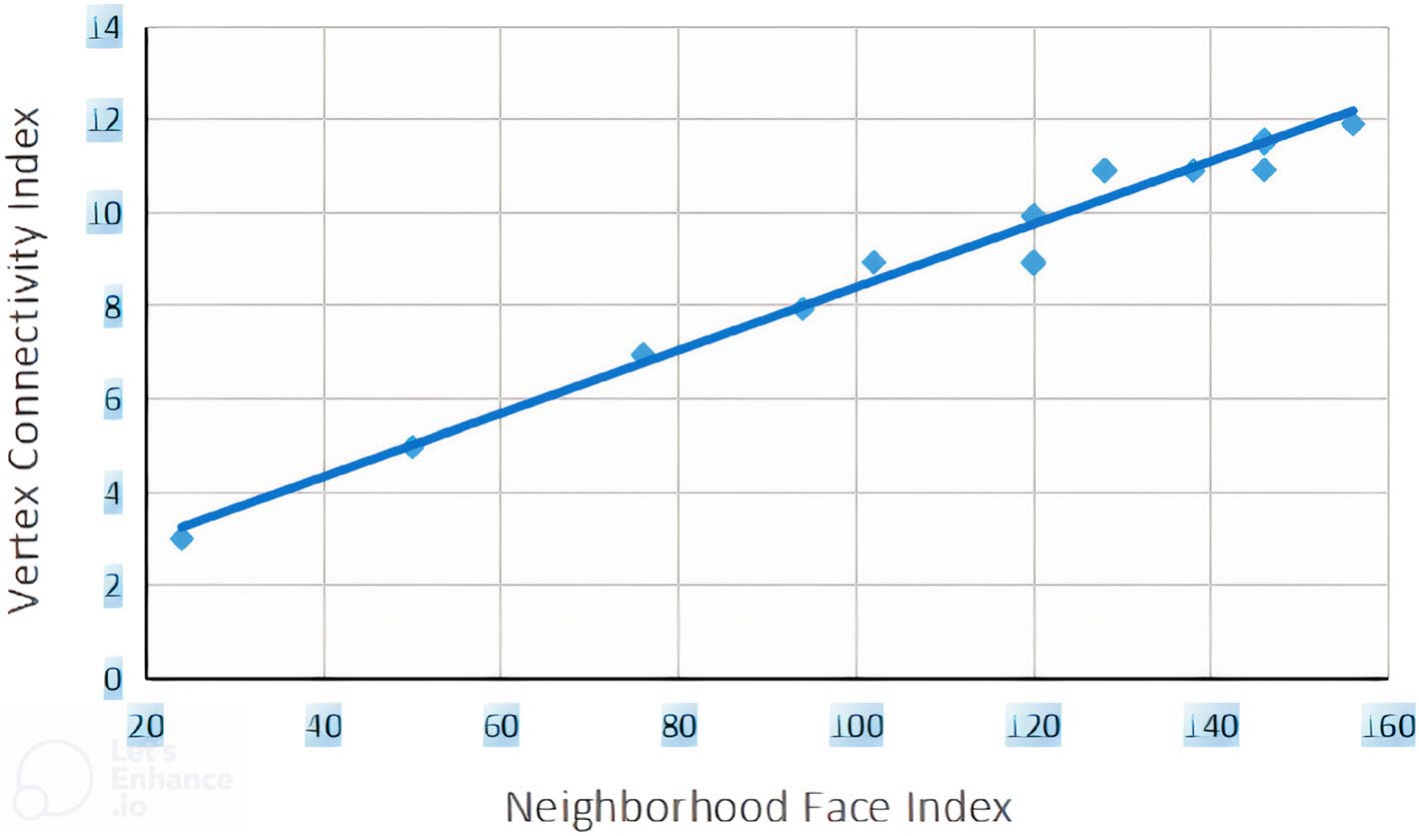
Scattered diagram of neighborhood face index versus vertex connectivity (randic) index.

**Figure 3:**
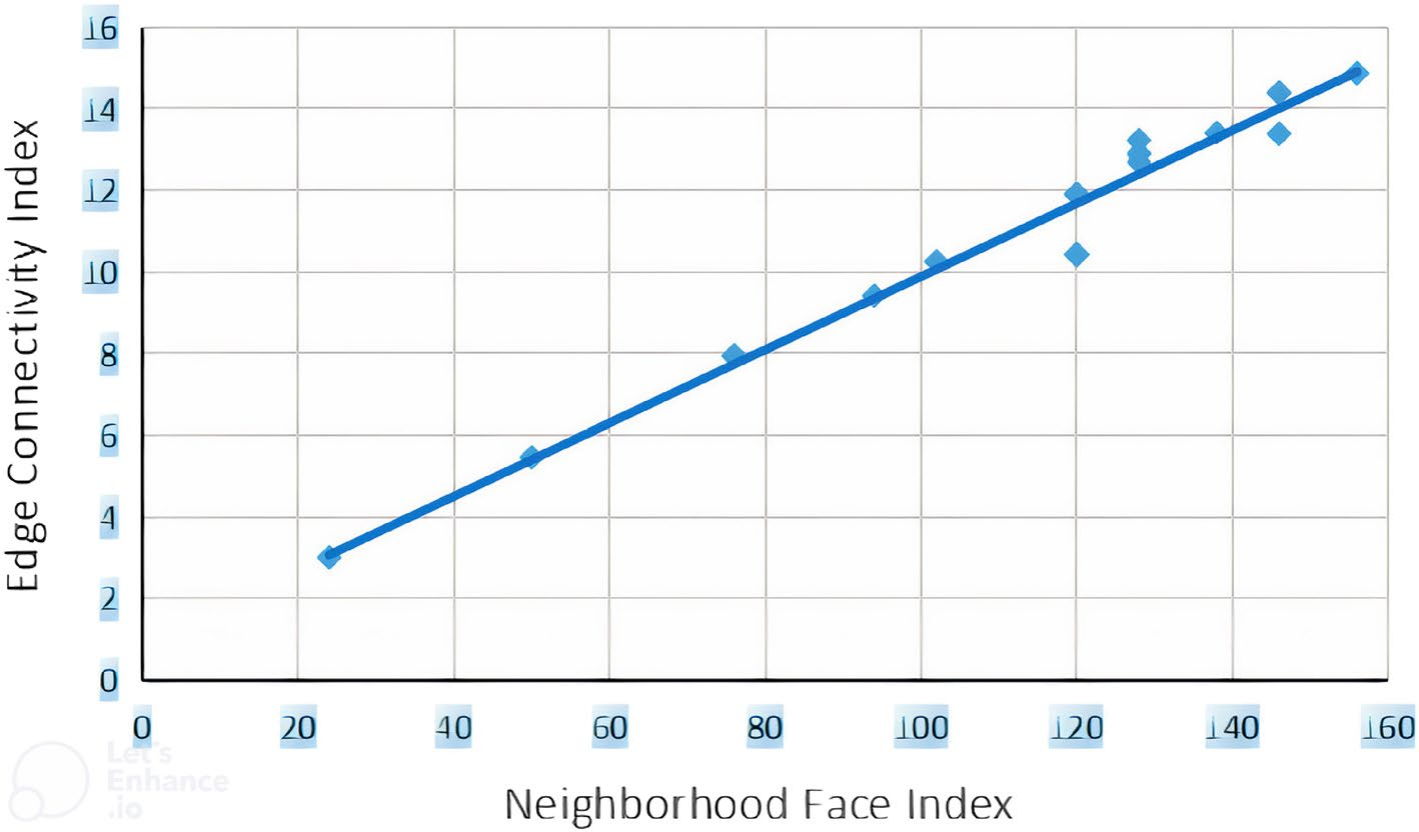
Scattered diagram of neighborhood face index and edge connectivity index.

The relationship between neighborhood face index and vertex connectivity index (randic index) is given in Eq. (1), while values for correlation coefficient *R*, adjusted squared correlation coefficient $R^2$, standard error of estimation *SEE*, Fisher ratio *F* and number of benzenoid hydrocarbons are also mentioned. We constructed statistical graphs for these correlations in Figs. 2 and 3, which are plotted for *NFI* versus *RI* and *NFI* versus *ECI*.

1 $$\begin{aligned} RI=0.025(\pm 0.01)NFI+2.288(\pm 0.290) \end{aligned}$$

$R=0.986$; $R^2(adjusted)=0.970$; $SEE=0.3982$; $F=695.844$; $n=21$ The relationship between neighborhood face index and edge connectivity index is mentioned in following equation

2 $$\begin{aligned} ECI=0.034(\pm 0.001)NFI+1.729(\pm 0.250) \end{aligned}$$

$$R=0.994$$; $$R^2(adjusted)=0.987$$; $$SEE=0.3438$$; $$F=1538.095$$; $$n=21$$.

### Linear regression model between NFI and E

**Figure 4:**
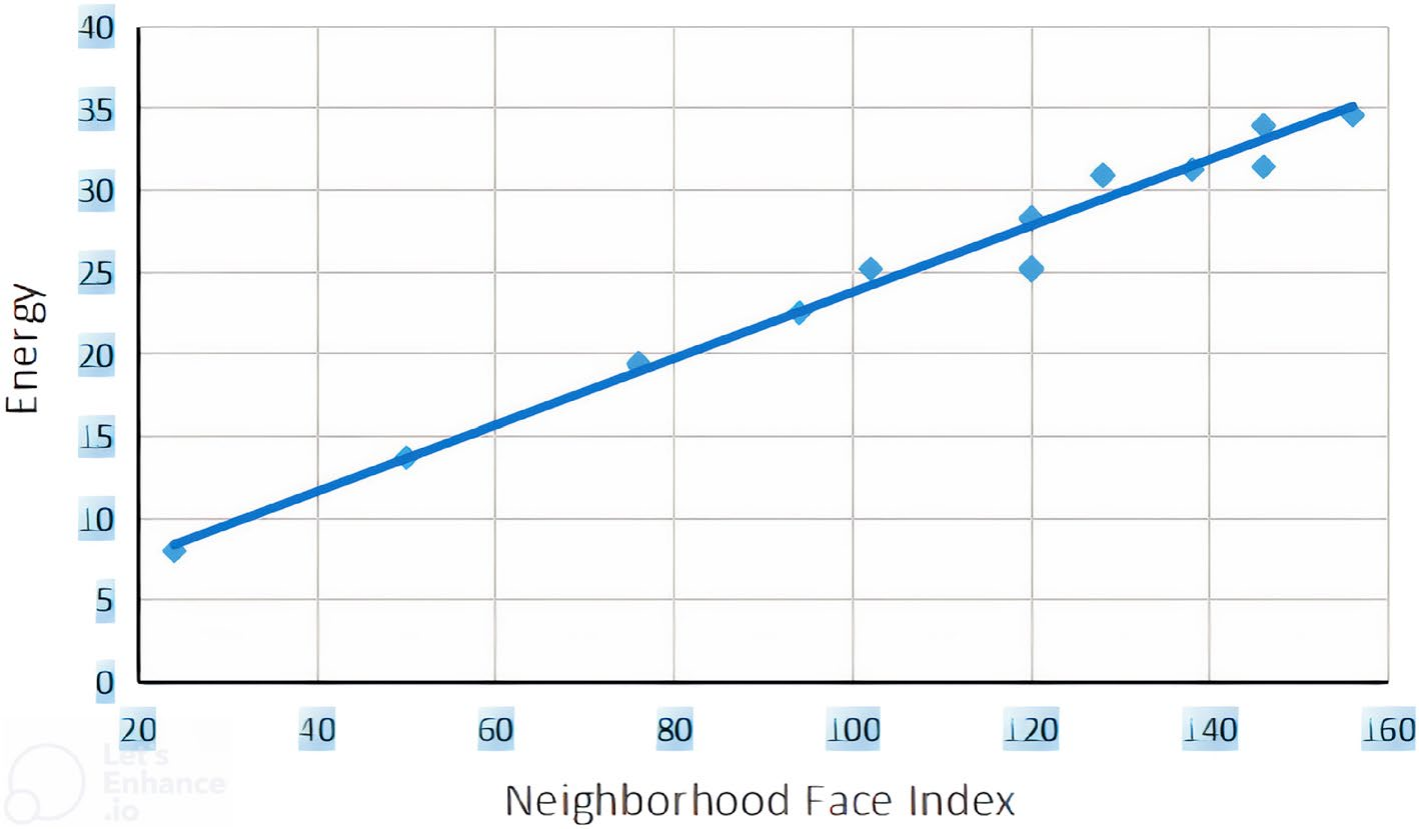
Scattered diagram of neighborhood face index and $\pi$-electron energy.

The linear regression models between neighborhood face index and $\pi -$electron energies of benzenoid hydrocarbons are constructed in Eq. (3) which shows a better correlation coefficient. Similarly, Eq. (4) describes the multiple linear regression model between $\pi$-electron energy, neghborhood face index, vertex connectivity index and edge connectivity index. We constructed statistical graphs for these correlations in Fig. 4, which is plotted between *NFI* versus $\pi$-electron energy (*E*).

3 $$\begin{aligned} E=0.076(\pm 0.002)NFI+5.435(\pm 0.7071) \end{aligned}$$

$$R=0.990$$; $$R^2(adjusted)=0.980$$; $$SEE=0.9741$$; $$F=987.512$$; $$n=21$$.

Multivariate correlation:

4 $$\begin{aligned} E=0.001(\pm 0.006)NFI+1.503(\pm 0.290)RI+1.085(\pm 0.336)ECI+0.120(\pm 0.3051) \end{aligned}$$

$$R=1.000$$; $$R^2(adjusted)=0.999$$; $$SEE=0.2015$$; $$F=7714.178$$; $$n=21$$.

### Linear regression model between NFI and BP

**Figure 5:**
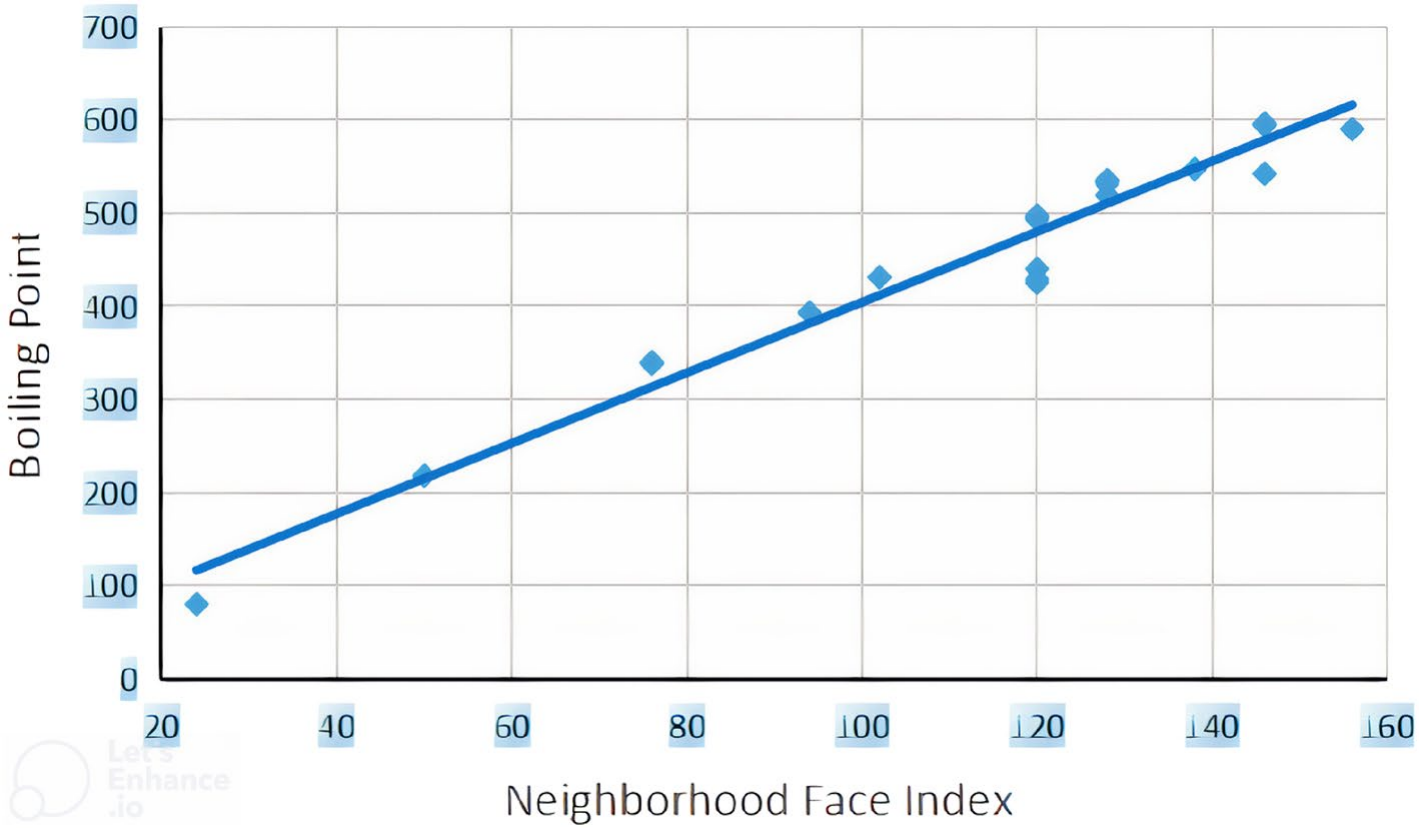
Scattered diagram of neighborhood face index and boiling points.

The linear regression models between neighborhood face index and boiling points of benzenoid hydrocarbons are constructed in Eq. (5) which shows a better correlation coefficient. Similarly, Eq. (6) describes the multiple linear regression model between boiling points, neghborhood face index, vertex connectivity index and edge connectivity index. We constructed statistical graphs for these correlations in Fig. 5, which is plotted between *NFI* versus boiling points *BP*.

5 $$\begin{aligned} BP=1.427(\pm 0.057)NFI+61.88(\pm 61.422) \end{aligned}$$

$$R=0.9994$$; $$R^2(adjusted)=0.969$$; $$SEE=22.5524$$; $$F=631.678$$; $$n=21$$. Multivariate correlation:

6 $$\begin{aligned} BP=-0.232(\pm 0.337)NFI+20.871(\pm 17.696)RI+33.201(\pm 20.494)ECI-43.263(\pm 18.600) \end{aligned}$$

$$R=0.996$$; $$R^2(adjusted)=0.991$$; $$SEE=12.303$$; $$F=723.172$$; $$n=21$$.

## Main results

**Figure 6:**
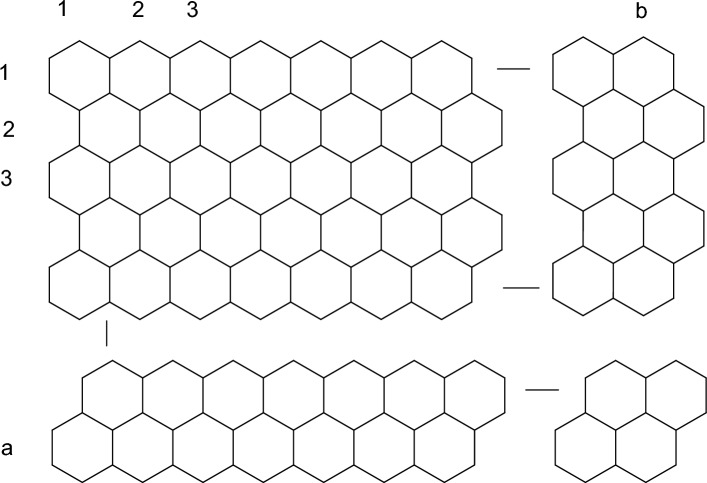
Two dimensional structure of graphene.

**Figure 7:**
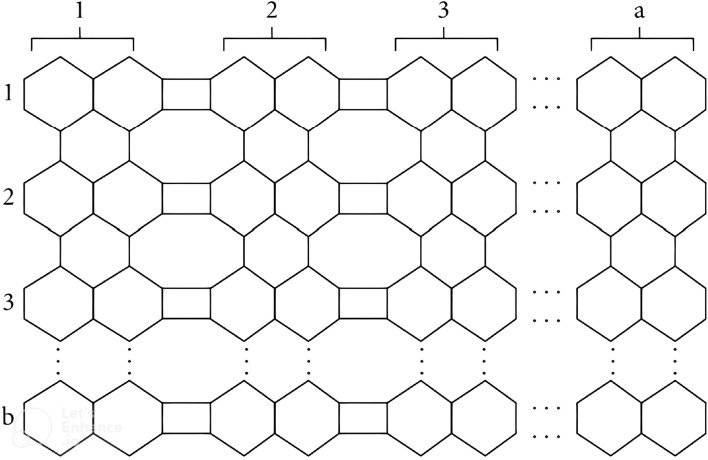
Two dimensional structure of H-naphthalenic nanosheet.

**Figure 8:**
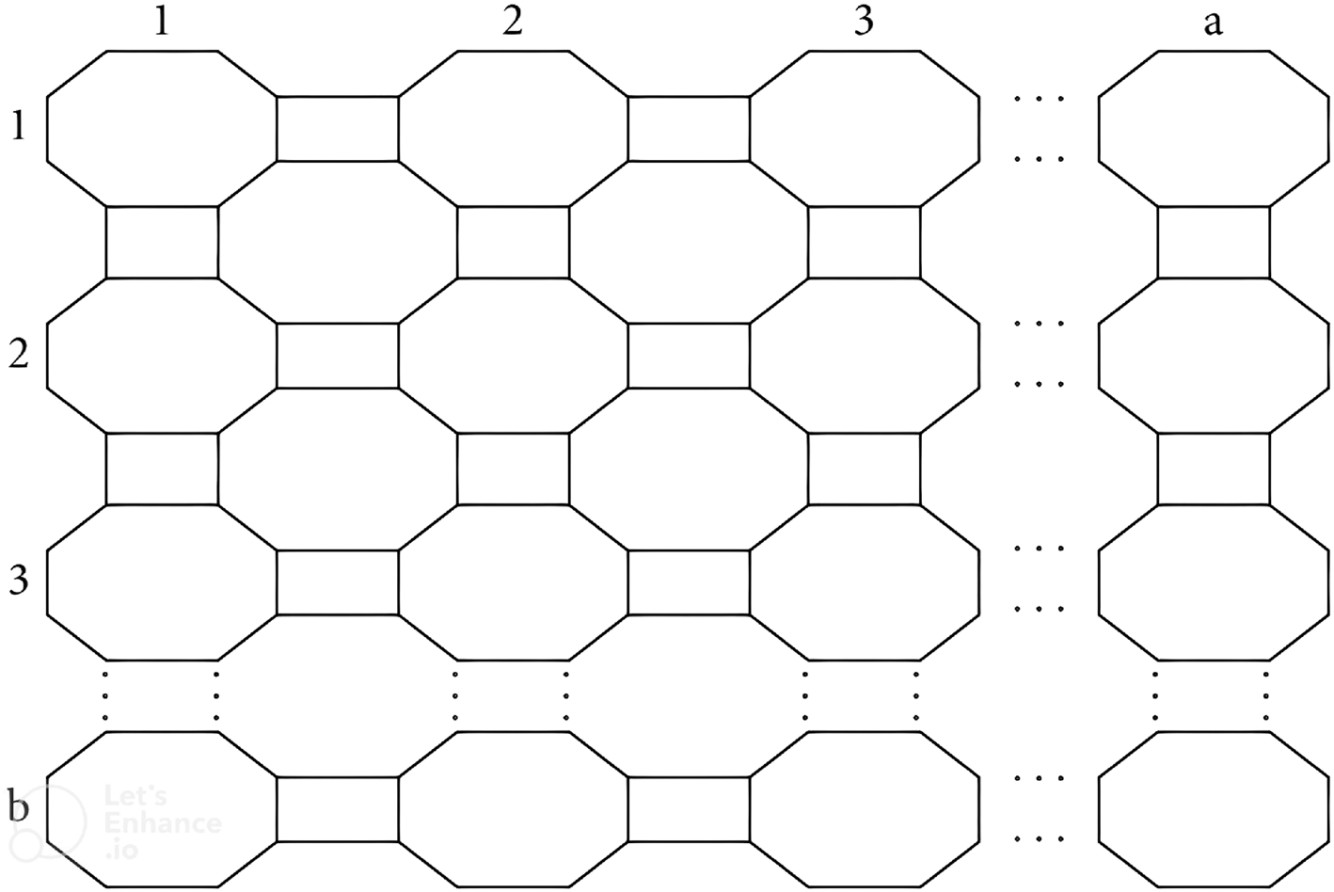
Two dimensional structure of $C_4C_8(S)$ nanosheet.

In this section, exact formulae for neighborhood face index of two dimensional Graphene, H-naphthalenic nanosheet and $C_4C_8(S)$ nanosheet structures are evaluated. Two dimensional chemical structures of these compounds are given in Figs. 6, 7 and 8, respectively. Then, computed results are examined using graphical analysis. The number of unit cells in each row are represented by *b* and number of rows are represented by *a*. Utilizing the frequencies of faces, we constructed the tables and computed required results.

### Result 1

Let *G* be the 2-dimensional molecular graph of graphene with $a,b \ge 1$. Then *NFI* of *G* is:

$$\begin{aligned} NFI(G) = \left\{ \begin{array}{ll} 14b-18&{}\quad for\; a=1\\ 54ab-30a+12b+142&{}\quad for \; a,b \ne 1\\ 14a-18 &{}\quad for \; b=1 \end{array} \right. \end{aligned}$$

### Proof

Two prove the required results, we partitioned two dimensional graphene structure containing a rows and b unit cells in following cases.

Case 1: Face index of hydro-benzenoid (1, 1) which is benzene molecule is 48. For, $a=1$ there exist two types of interval face $\pounds _{32}$ and $\pounds _{40}$ with cardinality 2 and $b-2$, respectively. The degree of external face is $2(13b-1)$. Then by definition, we have $NFI=14b-18$ for a = 1.

Case 1: For, $b=1$ there exist two types of interval face $\pounds _{32}$ and $\pounds _{40}$ with cardinality 2 and $a-2$, respectively. The degree of external face is $2(13a-1)$. Then by definition, we have $NFI=14a-18$ for b = 1.

Case 2: When $a,b \ne 1$, there are six types of enternal faces namely, $\pounds _{38}$, $\pounds _{42}$, $\pounds _{43}$, $\pounds _{47}$, $\pounds _{52}$, $\pounds _{54}$ and an external face $\pounds _{\infty }$. If $|\pounds _k|$ denotes the number of faces with neighborhood degree *k*, then following Table 2 represents the frequencies of such faces.

**Table 2:** Numbers of $\pounds _{38}$, $\pounds _{42}$, $\pounds _{43}$, $\pounds _{47}$, $\pounds _{52}$, $\pounds _{54}$ and $\pounds _{\infty }$ with given number of rows.

Rows	$|\pounds _{38}|$	$|\pounds _{42}|$	$|\pounds _{43}|$	$|\pounds _{47}|$	$|\pounds _{52}|$	$|\pounds _{54}|$	$d_n(\pounds _{\infty })$
(2,b)	2	2	2	2(b − 2)	0	0	26b + 24
(3,b)	2	2	2	2(b − 2)	1	b − 2	26b + 50
(4,b)	2	2	2	2(b − 2)	2	2(b − 2)	26b + 76
(5,b)	2	2	2	2(b − 2)	3	3(b − 2)	26b + 102
.	.	.	.	.	–	–	–
.	.	.	.	.	–	–	–
.	.	.	.	.	–	–	–
(a,b)	2	2	2	2(b − 2)	a − 2	(a − 2)(b − 2)	26(a + b) − 28

Utilizing the definition of neighborhood face index and Table 2

$$\begin{aligned} NFI(G)&=\sum _{\pounds \in F(G)}d_n(\pounds )=\sum _{ \pounds \in F(G)}d_n(\nu )\\&=\sum _{u\sim \pounds _{38}} d_n(u)+\sum _{u\sim \pounds _{42}} d_n(u)+\sum _{u\sim \pounds _{43}} d_n(u)+\sum _{u\sim \pounds _{47}} d_n(u)+\sum _{u\sim \pounds _{52}} d_n(u)\\&\quad +\sum _{u\sim \pounds _{54}} d_n(u)+\sum _{u\sim \pounds _{\infty }} d_n(u) \\&=38(2)+42(2)+43(2)+47(2b-4)+52(a-2)\\&\quad +54(a-2)(b-2)+26(a+b)-28\\&=54ab-30a+12b+142 \end{aligned}$$

which completes our proof. $\square$

### Result 2

Let *G* be the 2-dimensional molecular graph of H-naphthalenic nanosheet with $a,b \ge 1$. Then *NFI* of *G* is:

$$\begin{aligned} NFI(G) = \left\{ \begin{array}{ll} 186b-72 &{} \quad for\; a=1\\ 270ab-80a+60b-280 &{} \quad for \; a,b \ne 1\\ 190a-76&{} \quad for \; b=1 \end{array} \right. \end{aligned}$$

### Proof

Two prove the required results, we partitioned two dimensional H-naphthalenic nanosheet structure containing a rows and b unit cells in the following cases.

Case 1: For, $a=1$ there exist two types of interval face $\pounds _{32}$ and $\pounds _{42}$ with cardinality $(b+1)$ and $2(b-1)$, respectively. The degree of external face is $70b-20$. Then by definition, we have $NFI=186b-72$ for a = 1.

Case 2: For, $b=1$ there exist three types of interval face $\pounds _{38}$, $\pounds _{44}$ and $\pounds _{50}$ with cardinality 4, $2(a-2)$ and $(a-1)$, respectively. The degree of external face is $52a-21$. Then by definition, we have $NFI=190a-76$ for a = 1.

Case 3: When $a,b \ne 1$, there are eight types of internal faces namely, $\pounds _{34}$, $\pounds _{36}$, $\pounds _{38}$, $\pounds _{44}$, $\pounds _{48}$, $\pounds _{52}$, $\pounds _{54}$, $\pounds _{72}$ and an external face $\pounds _{\infty }$. If $|\pounds _k|$ denotes the number of faces with neighborhood degree *k*, then following Table 3 represents the frequencies of such faces. $\square$

**Table 3:** Numbers of $\pounds _{34}$, $\pounds _{36}$, $\pounds _{38}$, $\pounds _{44}$, $\pounds _{48}$, $\pounds _{52}$, $\pounds _{54}$, $\pounds _{72}$ and $\pounds _{\infty }$ with given number of rows.

Rows	$|\pounds _{34}|$	$|\pounds _{36}|$	$|\pounds _{38}|$	$|\pounds _{44}|$	$|\pounds _{48}|$	$|\pounds _{52}|$	$|\pounds _{54}|$	$|\pounds _{72}|$	$d_n(\pounds _{\infty })$
(2,b)	2(b-1)	0	4	0	4(b − 1)	2	b − 2	b − 1	70b + 32
(3,b)	2(b − 1)	b − 1	4	2	4(b − 1)	4	4b − 6	2(b − 1)	70b + 84
(4,b)	2(b − 1)	2(b − 1)	4	4	4(b − 1)	6	7b − 10	3(b − 1)	70b + 136
(5,b)	2(b − 1)	3(b − 1)	4	6	4(b − 1)	8	10b − 14	4(b − 1)	70b + 188
.	.	.	.	.	–	–	–	–	–
.	.	.	.	.	–	–	–	–	–
.	.	.	.	.	–	–	–	–	–
(a,b)	2(b − 1)	(a − 2)(b − 1)	4	2(a − 2)	4(b − 1)	2(a − 1)	3ab − 4a − 5b + 6	(a − 1)(b − 1)	52a + 70b − 72

$$\begin{aligned} NFI(G)&=\sum _{\pounds \in F(G)}d_n(\pounds )=\sum _{ \pounds \in F(G)}d_n(\nu )\\&=\sum _{u\sim \pounds _{34}} d_n(u)+\sum _{u\sim \pounds _{36}} d_n(u)+\sum _{u\sim \pounds _{44}} d_n(u)+\sum _{u\sim \pounds _{48}} d_n(u)+\sum _{u\sim \pounds _{52}} d_n(u)+\sum _{u\sim \pounds _{54}} d_n(u)\\&\quad +\sum _{u\sim \pounds _{72}} d_n(u)+\sum _{u\sim \pounds _{\infty }} d_n(u) \\&=34(2b-2)+36(a-2)(b-2)+38(4)+44(2a-4)+48(4b-4)+52(2a-2)\\&\quad +54(3ab-4a-5b+6)+72(a-1)(b-1)+52a+70b-72\\&=270ab-80a+60b-280 \end{aligned}$$

which completes our proof. $\square$

### Result 3

Let *G* be the 2-dimensional molecular graph of $C_4C_8(S)$ nanosheet with $a,b \ge 1$. Then *NFI* of *G* is:

$$\begin{aligned} NFI(G) = \left\{ \begin{array}{ll} 136b-72&{} \quad for\; a=1\\ 216ab -80(a+b)-64 &{} \quad for \; a,b \ne 1\\ 136a-72 &{} \quad for \; b=1 \end{array} \right. \end{aligned}$$

### Proof

Two prove the required results, we partitioned two dimensional $C_4C_8(S)$ nanosheet structure containing a rows and b unit cells in following cases.

Case 1: For, $a=1$ there exist three types of interval face $\pounds _{32}$, $\pounds _{42}$ and $\pounds _{52}$ with cardinality $b-1$, 2 and $b-2$, respectively. The degree of external face is $52b-20$. Then by definition, we have $NFI=136b-72$ for $a=1$.

Case 2: For, $b=1$ there exist three types of interval face $\pounds _{32}$, $\pounds _{42}$ and $\pounds _{52}$ with cardinality $a-1$, 2 and $a-2$, respectively. The degree of external face is $52a-20$. Then by definition, we have $NFI=136a-72$ for $b=1$.

Case 3: When $a,b \ne 1$, there are five types of enternal faces namely, $\pounds _{34}$, $\pounds _{36}$, $\pounds _{52}$, $\pounds _{62}$, $\pounds _{72}$ and an external face $\pounds _{\infty }$. If $|\pounds _k|$ denotes the number of faces with neighborhood degree *k*, then following Table 4 represents the frequencies of such faces.

**Table 4:** Numbers of $\pounds _{34}$, $\pounds _{36}$, $\pounds _{52}$, $\pounds _{62}$, $\pounds _{72}$, and $\pounds _{\infty }$ with given number of rows.

Rows	$|\pounds _{34}|$	$|\pounds _{36}|$	$|\pounds _{52}|$	$|\pounds _{62}|$	$|\pounds _{72}|$	$d_n(\pounds _{\infty })$
(2,b)	2b	b − 2	4	2(b − 2)	b − 1	52b + 32
(3,b)	2(b + 1)	3b − 5	4	2(b − 1)	3b − 4	52b + 84
(4,b)	2(b + 2)	5b − 8	4	2(b)	5b − 7	52b + 136
(5,b)	2(b − 3)	7b − 11	4	2(b + 1)	7b − 10	52b + 188
.	.	.	.	–	–	–
.	.	.	.	–	–	–
.	.	.	.	–	–	–
(a,b)	2(a + b − 2)	2ab − 3a − 3b + 4	4	2(a + b − 4)	2ab − 3a − 3b + 4	52(a + b) − 72

$$\begin{aligned} NFI(G)&=\sum _{\pounds \in F(G)}d_n(\pounds )=\sum _{ \pounds \in F(G)}d_n(\nu )\\&=\sum _{u\sim \pounds _{34}} d_n(u)+\sum _{u\sim \pounds _{36}} d_n(u)+\sum _{u\sim \pounds _{52}} d_n(u)+\sum _{u\sim \pounds _{62}} d_n(u)+\sum _{u\sim \pounds _{72}} d_n(u)+\sum _{u\sim \pounds _{\infty }} d_n(u) \\&=34(2a+2b-4)+36(2ab-3a-3b+4)+52(4)+62(2a+2b-8)\\&\quad +72(2ab-3a-3b+4)+52(a+b)-72\\&= 216ab -80(a+b)-64 \end{aligned}$$

which completes our proof. $\square$

### Result 4

Let *G* be the 2-dimensional molecular graph of $C_4C_8(R)$ nanosheet with $a,b \ge 1$. Then *NFI* of *G* is:

$$\begin{aligned} NFI(G) = \left\{ \begin{array}{ll} 174b+98 &{} \quad for\; a=1\\ 108ab+66 a+246b-472 &{} \quad for \; a,b \ne 1\\ 174a+98 &{} \quad for \; b=1 \end{array} \right. \end{aligned}$$

**Figure 9:**
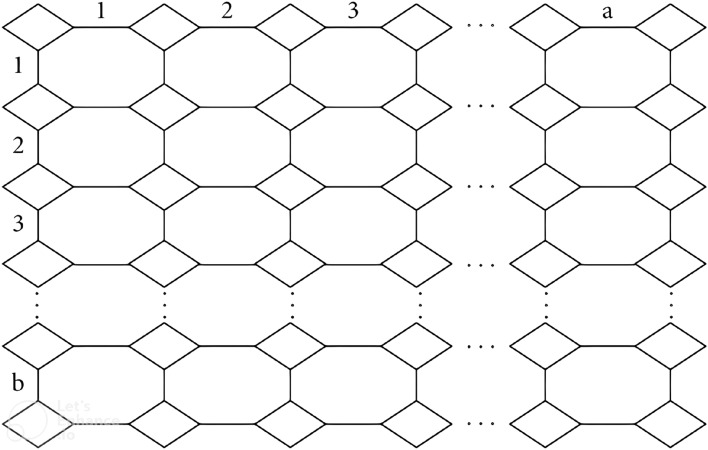
Two dimensional structure of $C_4C_8(R)$ nanosheet.

### Proof

Two prove the required results, we partitioned two dimensional $C_4C_8(R)$ nanosheet structure as given in Fig. 9 containing a rows and b unit cells in the following cases.

Case 1: For, $a=1$ there exist four types of interval face $\pounds _{26}$, $\pounds _{31}$, $\pounds _{66}$ and $\pounds _{68}$ with cardinality 4, $2(b-1)$, 2 and $(b-2)$, respectively. The degree of external face is $44b+60$. Then by definition, we have $NFI=186b-72$ for a = 1.

Case 2: For, $b=1$ there exist four types of interval face $\pounds _{26}$, $\pounds _{31}$, $\pounds _{66}$ and $\pounds _{68}$ with cardinality 4, $2(a-1)$, 2 and $(a-2)$, respectively. The degree of external face is $44a+60$. Then by definition, we have $NFI=186a-72$ for b = 1.

Case 3: When $a,b \ne 1$, there are six types of internal faces namely, $\pounds _{26}$, $\pounds _{31}$, $\pounds _{36}$, $\pounds _{68}$, $\pounds _{70}$, $\pounds _{72}$ and an external face $\pounds _{\infty }$. If $|\pounds _k|$ denotes the number of faces with neighborhood degree *k*, then following Table 5 represents the frequencies of such faces.

**Table 5:** Numbers of $\pounds _{26}$, $\pounds _{31}$, $\pounds _{36}$, $\pounds _{68}$, $\pounds _{70}$, $\pounds _{72}$ and $\pounds _{\infty }$ with given number of rows.

Rows	$|\pounds _{26}|$	$|\pounds _{31}|$	$|\pounds _{36}|$	$|\pounds _{68}|$	$|\pounds _{70}|$	$|\pounds _{72}|$	$d_n(\pounds _{\infty })$
(2,b)	4	2b	(b − 1)	4	2(b − 2)	0	44b + 104
(3,b)	4	2(b + 1)	2(b − 1)	4	2(b − 1)	(b − 2)	44b + 148
(4,b)	4	2(b + 2)	3(b − 1)	4	2(b − 0)	2(b − 2)	44b + 192
(5,b)	4	2(b + 3)	4(b − 1)	4	2(b + 1)	3(b − 2)	44b + 236
.	.	.	.	.	–	–	–
.	.	.	.	.	–	–	–
.	.	.	.	.	–	–	–
(a,b)	4	(a + b − 2)	(a − 1)(b − 1)	4	2(a + b − 4)	(a − 2)(b − 2)	44(a + b) + 16

$$\begin{aligned} NFI(G)&=\sum _{\pounds \in F(G)}d_n(\pounds )=\sum _{ \pounds \in F(G)}d_n(\nu )\\&=\sum _{u\sim \pounds _{26}} d_n(u)+\sum _{u\sim \pounds _{31}} d_n(u)+\sum _{u\sim \pounds _{36}} d_n(u)+\sum _{u\sim \pounds _{68}} d_n(u)+\sum _{u\sim \pounds _{70}} d_n(u)\\&\quad +\sum _{u\sim \pounds _{72}} d_n(u)+\sum _{u\sim \pounds _{\infty }} d_n(u) \\&=26(4)+62(a+b-2)+36(a-1)(b-1)+68(4)+140(a+b-4)\\&\quad +72(a-2)(b-2)+44(a+b)+16 \\&=108ab+66a+246b-472 \end{aligned}$$

which completes our proof. $\square$

## Graphical analysis

**Figure 10:**
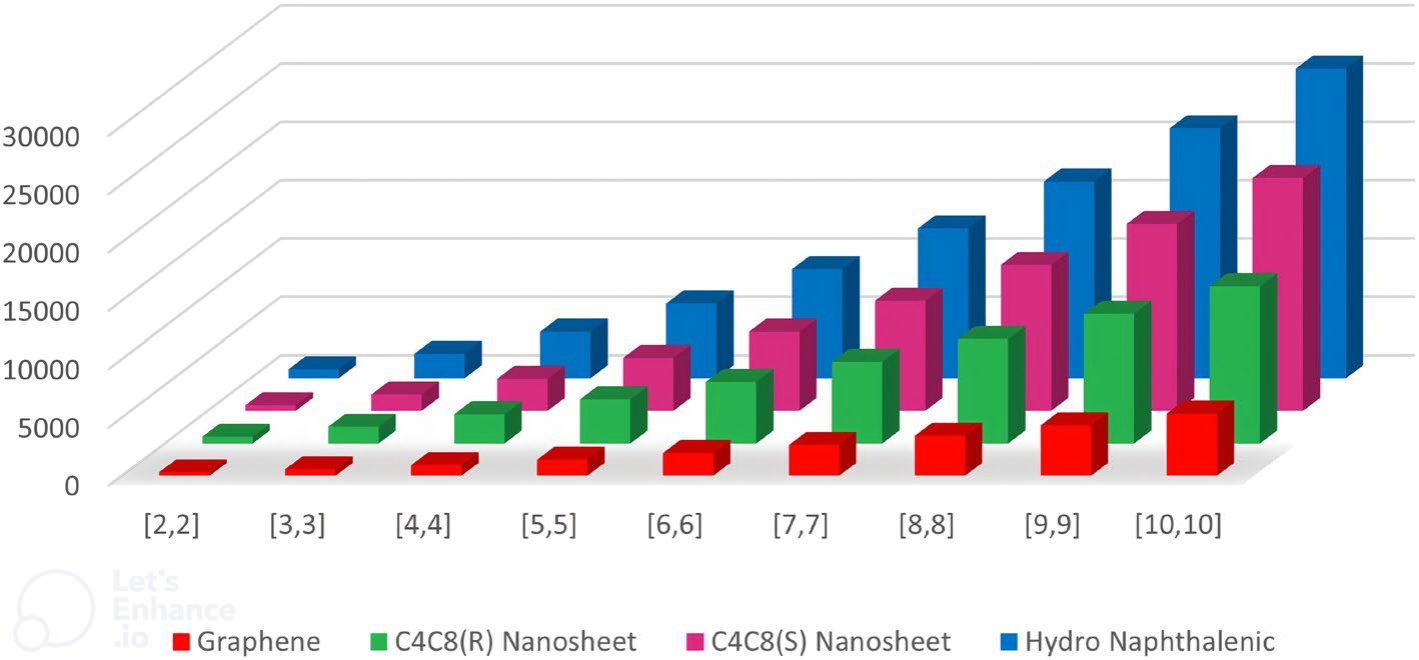
Neighborhood face index for (i) Graphene (ii) H-naphthalenic (iii) $C_4C_8(S)$ and (iv) $C_4C_8(R)$ nanosheets.

The graphical representation depicted in Fig. 10 offers a visual insight into the evolving characteristics of topological descriptors as the number of molecules within a chemical structure increases. These figures vividly illustrate how these descriptors change with the expansion of the molecular set, providing a valuable perspective on structural trends. For a more detailed examination of these changes, Table 6 presents the numeric values of the Neighborhood Face Index (NFI). The data in this table reveals a discernible and progressive rise in the calculated NFI values, corresponding to the growth of the chemical structures. This observation underscores the relationship between molecular complexity and the NFI, shedding light on the structural intricacies that emerge as the chemical structure becomes more elaborate.

**Table 6:** Neighborhood face index for graphene, H-naphthalenic, $C_4C_8(S)$ and $C_4C_8(R)$ nanosheets assuming a=b for different values.

$$\begin{array}{c} [a,b] \end{array}$$	NFI of graphene nanosheet	NFI of hydro naphthalenic nanosheet	NFI of $C_4C_8(S)$	NFI of $C_4C_8(R)$ nanosheet
[2, 2]	322	760	480	584
[3, 3]	574	2090	1400	1436
[4, 4]	934	3960	2752	2504
[5, 5]	1402	6370	4536	3788
[6, 6]	1978	9320	6752	5288
[7, 7]	2662	12,810	9400	7004
[8, 8]	3454	16,840	12,480	8936
[9, 9]	4354	21,410	15,992	11,084
[10, 10]	5362	26,520	19,936	13,448

## Conclusions

In this article, we introduced a new topological invariant namely, neighborhood face index which exhibits an extraordinary correlation coefficient $R \ge 0.999$ for boiling points and $R \ge 0.990$ for $\pi$-electron energies of benzenoid hydrocarbons utilizing regression models of *NFI* with mentioned physio-chemical quantities. We also calculated exact values of newly introduced TI for some carbon nanosheets and analyzed obtained results graphically to understand their behavior with variation in molecular structure as shown in Fig. 10. Our research work motivates researchers to examine the behaviour of different chemical compounds utilizing *NFI*.

## Data Availability

The paper includes the information used to verify the study’s findings.
